# Comparative assessment of autochthonous bacterial and fungal communities and microbial biomarkers of polluted agricultural soils of the Terra dei Fuochi

**DOI:** 10.1038/s41598-018-32688-5

**Published:** 2018-09-24

**Authors:** Valeria Ventorino, Alberto Pascale, Paola Adamo, Claudia Rocco, Nunzio Fiorentino, Mauro Mori, Vincenza Faraco, Olimpia Pepe, Massimo Fagnano

**Affiliations:** 1 Department of Agricultural Sciences, Division of Microbiology, University of Naples Federico II, Via Università 100, Portici, 80055 Naples Italy; 2 Department of Agricultural Sciences, Division of Agricultural Chemistry and Pedology, University of Naples Federico II, Via Università 100, Portici, 80055 Naples Italy; 3 Department of Agricultural Sciences, Division of Plant Biology and Crop Science, University of Naples Federico II, Via Università 100, Portici, 80055 Naples Italy; 4 Department of Chemical Sciences, University of Naples Federico II, Complesso Universitario Monte S. Angelo, Naples, 80126 Italy; 5 Task Force on Microbiome Studies, University of Naples Federico II, Naples, Italy

## Abstract

Organic and inorganic xenobiotic compounds can affect the potential ecological function of the soil, altering its biodiversity. Therefore, the response of microbial communities to environmental pollution is a critical issue in soil ecology. Here, a high-throughput sequencing approach was used to investigate the indigenous bacterial and fungal community structure as well as the impact of pollutants on their diversity and richness in contaminated and noncontaminated soils of a National Interest Priority Site of Campania Region (Italy) called “Terra dei Fuochi”. The microbial populations shifted in the polluted soils via their mechanism of adaptation to contamination, establishing a new balance among prokaryotic and eukaryotic populations. Statistical analyses showed that the indigenous microbial communities were most strongly affected by contamination rather than by site of origin. Overabundant taxa and Actinobacteria were identified as sensitive biomarkers for assessing soil pollution and could provide general information on the health of the environment. This study has important implications for microbial ecology in contaminated environments, increasing our knowledge of the capacity of natural ecosystems to develop microbiota adapted to polluted soil in sites with high agricultural potential and providing a possible approach for modeling pollution indicators for bioremediation purposes.

## Introduction

In recent decades, widespread environmental multicontamination with organic (i.e., polycyclic aromatic hydrocarbons, petroleum and related products) and inorganic (i.e., potentially toxic elements) pollutants due to urban, industrial and agricultural activities as well as illegal toxic waste dumping has posed a huge threat to human health and natural ecosystems^1^.

Soil contamination generally affects the potential ecological function of the environment, altering soil functioning, health and biodiversity^2^, contributing to most of the soil degradation in terms of its microbial abundance and diversity^3^. In fact, soil pollution could cause pressure on sensitive microorganisms and thus could change the composition of microbial community^4^. Although organic pollutants have been shown to reduce microbial biodiversity^5^, they can be used as a carbon source by some species of microorganisms^6^, thereby stimulating their growth in contaminated soil and thus leading to the development of a new microbial community diversity^7^. However, little is known about the microbial response to multicontamination and remediation practices due to the high biodiversity of microflora as well as to the complex relationships among microbial communities and biotic and abiotic processes influencing their activities in soil^8^. Therefore, investigating and understanding the interactions between microorganisms and soil components will assist us in exploring and establishing the potential relevance between soil microorganisms and microbial processes^9^. The use of novel approaches based on culture-independent high-throughput sequencing can reveal uncultivable microbiota and enables the study of microbial ecology and taxonomic diversity at a high resolution^10^, allowing a broader range of comparisons between different soils with varying levels of contaminants. Moreover, this approach could also be useful for identifying specific microbial biomarkers that could be used as indicators for the ecological *status* and health of soils as well as for possible biotechnological applications for bioremediation plans. Various studies have been conducted to investigate the impact of contamination on the indigenous microbial populations and their shift in order to discover phylogenetic markers with potential degradative abilities. These studies revealed different microbial populations organized in complex communities on the basis of the environmental conditions. In particular, contrasting observations of the impact of contaminants on microbial diversity due to many factors that are involved in the microbial response to pollutants have been reported^11^. Proteobacterial populations were dominant and recurrent in petroleum- and polycyclic aromatic hydrocarbons (PAHs) -polluted soils^12,13^ and in coastal sediments^14^ as well as in uranium mines^15,16^. Fragoso dos Santos *et al*.^17^ indicated possible targets for the biomonitoring of the impact of oil in mangroves. The order Chromatiales and the genus *Haliea* were detected as sensitive indicators, while the three genera *Marinobacterium*, *Marinobacter* and *Cycloclasticus* were reported as resistant taxa. Jeanbille *et al*.^18^ identified prokaryotic and eukaryotic potential biomarkers for PAH chronic contamination in coastal sediments. Additionally, the history of pollution plays a crucial role in the microbial community structure. In fact, no general trend has emerged yet, and the short-term impact of contamination tends to decrease microbial abundance, richness and diversity, while in aged or chronically contaminated environments, a surprisingly high bacterial diversity, due to adaptation over time and stability caused by long-term exposure, has been observed^7,18^.

In this context, the aim of this study was to determine and describe the native microbiota and the impact of anthropogenic pollution (mainly by heavy hydrocarbons but in some cases also by copper and zinc) on the diversity and richness of prokaryotic and eukaryotic communities occurring in soils of two rural sites of Campania (southern Italy) subject to illicit waste disposal and dumping or suspected to be polluted by metals due to agricultural practices. Both these sites are located in an area formerly classified as National Interest Priority Sites (NIPS)^19^ and are actually identified by the Italian State as Regional Interest Priority Sites (RIPS)^20^.

## Results and Discussion

### Characteristics of the sampling sites

The studied soils were characterized by very similar chemical properties and particle size distribution.

Both soils were sandy loam, with 61% (in Giugliano, G) and 71% (in Trentola Ducenta, TD) of the soil in the sand fraction. Soil pH-H_2_O was neutral in G (pH 7.3 ± 0.2) and subalkaline in TD (pH 8.0 ± 0.2), in agreement with the carbonate content (8 ± 2.5 g kg^−1^ in G *vs*. 174 ± 41 g kg^−1^ in TD). The electrical conductivity was always below the values that limit plant growth and agricultural production (0.16 ± 0.03 and 0.45 ± 0.3 dS m^−1^ in G and TD, respectively). The soil organic carbon content was never above 2%, with similar values in both sites (G: 20 ± 3.1 g kg^−1^; TD: 18 ± 0.7 g kg^−1^). The cation exchange capacity (CEC) was 28 ± 3.1 cmol(+) kg^−1^ in TD and 34 ± 3.2 cmol(+) kg^−1^ in G, with a dominance (~80%) of calcium in the exchange complex. Therefore, the physical-chemical fertility of both the soils did not result in limiting plant growth and did not show any features that may alter the microbial conditions.

It was found that the soils of four on seven plots for both G and TD sites can be considered potentially contaminated for residential use, in accordance with Italian environmental law (Law Decree 152/2006) (Table 1). The analysis of the “pseudototal” concentration of potentially toxic elements (PTEs) showed that Cu and Zn were the only such elements occurring in the soil samples in amounts above the Italian thresholds of 120 and 150 mg kg^−1^, respectively. Likewise, the concentration of heavy hydrocarbons (C > 12) in both G and TD soils was well above the Italian threshold of 50 mg kg^−1^, while for PAHs, only benzo(a)pyrene was found, in few cases, at concentrations equal to or slightly above the threshold of 0.10 mg kg^−1^.

**Table 1:** Organic and inorganic pollutant concentration (mg kg^−1^) in soil samples collected from Giugliano (GI) and Trentola-Ducenta (TD) pilot sites.

Sample	C > 12	Benzo(a)pyrene	Cu	Zn	Typology
G 6-3	36	0.03	99	76	Noncontaminated
G 8-3	43	0.0005*	50	71	Noncontaminated
G 8-6	48	0.03	48	67	Noncontaminated
Mean ± SD	42 ± 6.0	0.02 ± 0.02	66 ± 28.9	71 ± 4.5	
G 1-3	533	0.04	219	170	Contaminated
G 8-2	705	0.03	91	80	Contaminated
G 8-5	401	0.03	96	128	Contaminated
G 8-8	590	0.13	53	79	Contaminated
Mean ± SD	557 ± 126	0.06 ± 0.06	80 ± 24	96 ± 28	
TD 4-1	37	0.0005^*^	65	64	Noncontaminated
TD 4-5	31	0.0005^*^	86	103	Noncontaminated
TD 4-7	36	0.0005^*^	75	69	Noncontaminated
Mean ± SD	35 ± 3.2	0.001 ± 0.00	75 ± 11	79 ± 21	
TD 21-9	206	0.07	75	125	Contaminated
TD 32-4	329	0.05	51	114	Contaminated
TD 32-7	541	0.04	42	228	Contaminated
TD 32-8	250	0.05	56	98	Contaminated
Mean ± SD	332 ± 149	0.05 ± 0.01	50 ± 7.1	147 ± 71	
**Italian thresholds (D.Lgs 152/2006)**	**50**	**0.10**	**120**	**150**	

^*^Value below detection limit (BDL), to assess the mean it was used the DL/2.

Table 1 presents the single amounts of heavy hydrocarbons, benzo(a)pyrene, Cu and Zn in the polluted and nonpolluted plots in G and TD. Four of seven plots in both Giugliano and Trentola Ducenta were contaminated by C > 12 (G: mean 557 ± 126 mg kg^−1^, range 401–705 mg kg^−1^; TD: mean 332 ± 149 mg kg^−1^, range 206–541 mg kg^−1^), Cu (G: mean 115 ± 72 mg kg^−1^, range 53–219 mg kg^−1^; TD: mean 56 ± 14 mg kg^−1^, range 42–75 mg kg^−1^) and Zn (G: mean 114 ± 44 mg kg^−1^, range 79–170 mg kg^−1^; TD: mean 141 ± 59 mg kg^−1^, range 98–228 mg kg^−1^), while the remaining 6 plots (3 in G and 3 in TD) were considered not contaminated.

### Microbial community diversity

The microbial diversity from G and TD was characterized by partial 16S and 18S rRNA gene sequencing obtained from DNA directly extracted from soil samples of noncontaminated (NoCont) and long-term contaminated (Cont) plots. In total, 4,613,050 and 1,226,128 high quality reads were analyzed for prokaryotes and eukaryotes, respectively. The alpha-diversity was determined by calculating the Shannon diversity index and the Chao1 richness index based on OTUs of 97% identity.

**Figure 1:**
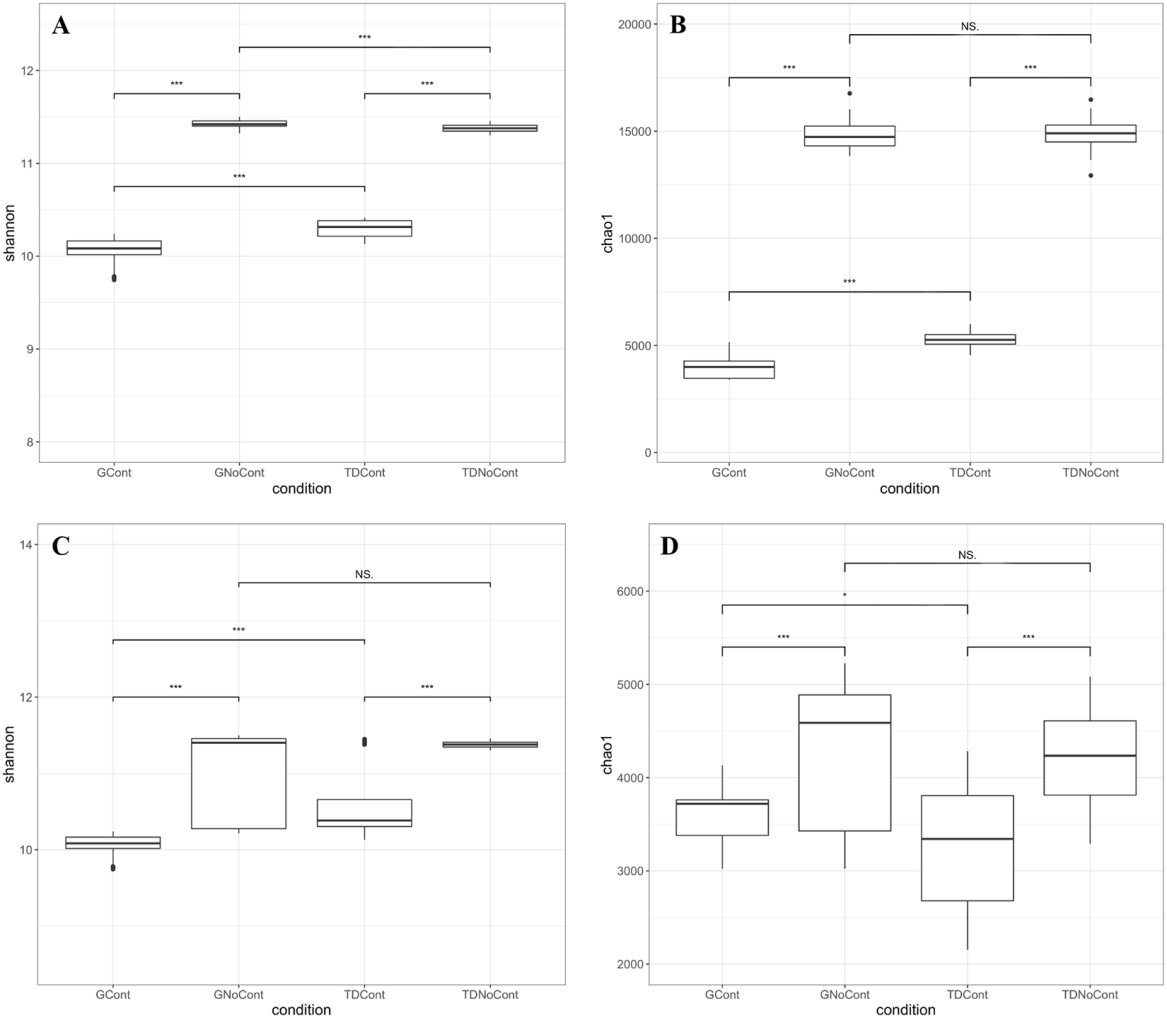
Box plots showing Shannon diversity and Chao1 richness indices based on prokaryotic (**A**,**B**) and eukaryotic (**C**,**D**) communities in the soil samples. Boxes represent the interquartile range (IQR) between the first and third quartiles, and the line inside represents the median (2nd quartile). Whiskers denote the lowest and the highest values within 1.5 × IQR from the first and third quartiles, respectively. Asterisks indicate a significant difference as obtained by pairwise Wilcoxon test (^*^p < 0.05; ^**^p < 0.01; ^***^p < 0.001). NS denote not significant difference. GCont: contaminated soils at Giugliano site; GNoCont: noncontaminated soils at Giugliano site; TDCont: contaminated soils at Trentola Ducenta site; TDNoCont: noncontaminated soils at Trentola Ducenta site.

As shown in Fig. 1, in both sites, strong differences in prokaryotic and eukaryotic diversity were found between noncontaminated and contaminated soils, as revealed by the Shannon and Chao1 indexes, whereas the native microbiota was similar between the G and TD sites. In particular, microbial diversity and richness were lower in contaminated plots than noncontaminated control plots, highlighting a significant association between the contaminants and the microbial diversity (GCont *vs*. GNoCont and TDCont *vs*. TDNoCont, *P* < 0.001). The presence of environmental stressors such as heavy metals strongly reduced the total bioactivity, richness and diversity of microorganisms with increasing pollutant concentrations in the soil^21,22^.

By comparison of G and TD sites, significant differences in bacterial diversity were observed between contaminated plots, as indicated by both Shannon and Chao1 (GCont *vs*. TDCont, *P* < 0.001) indexes (Fig. 1A,B). A similar result was obtained in noncontaminated plots (GNoCont *vs*. TDNoCont, *P* < 0.001) with the Shannon index (Fig. 1A), although no significant difference in richness based on the Chao1 index was observed (Fig. 1B).

The same behavior was shown by fungal and oomycetal diversity (*P* < 0.001) and richness (*P* < 0.05) when comparing GCont and TDCont samples (Fig. 1C,D), while no differences were detected between noncontaminated soils (GNoCont *vs*. TDNoCont, Fig. 1C,D). These results could be due to selective pressure exerted by pollutants on microbiota in contaminated soils regardless of the site of origin of the samples. Yao *et al*.^23^ reported that although the microbial diversity was reduced in contaminated soils, resistant microbial populations were enhanced. In addition, the occurrence of metal-tolerant microbes increased with increasing heavy metal concentrations in polluted sites^24^.

**Figure 2:**
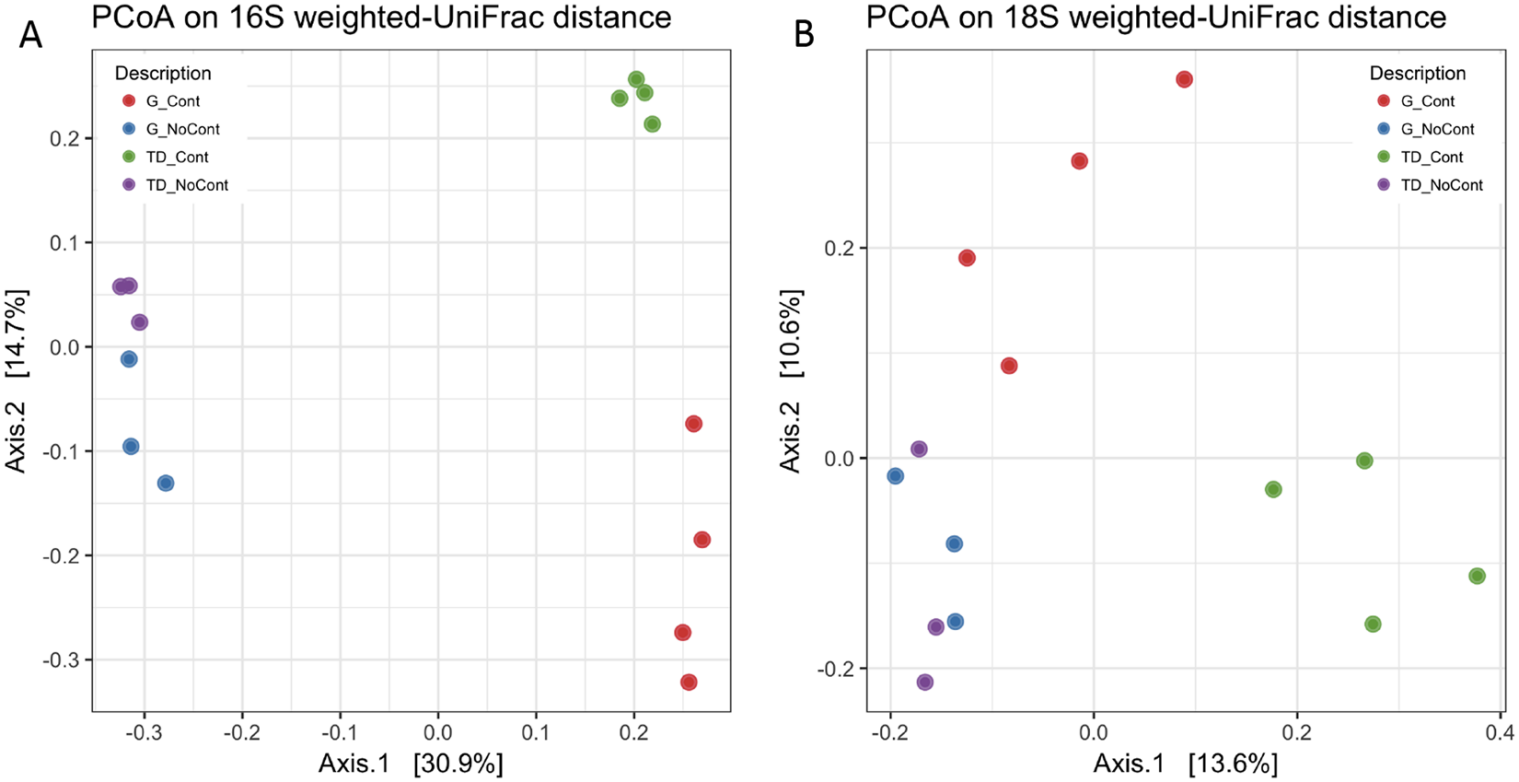
Principal Coordinates Analysis of weighted UniFrac distances for 16S (**A**) and 18S (**B**) rRNA gene sequence data of Giugliano and Trentola Ducenta soil samples. GCont (red): contaminated soils at Giugliano site; GNoCont (blue): noncontaminated soils at Giugliano site; TDCont (green): contaminated soils at Trentola Ducenta site; TDNoCont (violet): noncontaminated soils at Trentola Ducenta site.

As shown in Fig. 2, the PCoA of the weighted UniFrac community distances showed a marked difference between the microbiota of contaminated and noncontaminated soil samples, especially for the bacterial communities. In fact, the samples of both G and TD unpolluted soils grouped separately on the left side of the chart in Fig. 2A compared to contaminated soils. The contaminated samples underwent selective pressure due to the occurrence of xenobiotic compounds, showing an evident separation on PCoA chart between unpolluted and polluted G and TD soils as well as between polluted samples (GCont at the bottom right of the chart and TDCont at the top right) (Fig. 2A). This behavior could be due to the different adaptation mechanisms used by several microbial groups to survive and grow under these stress conditions. A similar trend was observed for the eukaryotic populations, although the distribution of the samples was less marked (Fig. 2B). Moreover, the statistical test ANOSIM showed that the composition of bacterial and fungal communities in the analyzed soils was significantly influenced by contamination (*P* < 0.01). This difference increased when the two factors were combined. In fact, ANOSIM showed a significant difference for *site-origin* x *contamination* (*P* < 0.001), demonstrating a correlation between the contamination and the site of origin of samples.

### Microbial taxonomic composition

Relative abundances of bacterial and fungal taxa were examined at the phyla and class level to determine whether there were any significant shifts in the composition of the microbial communities according to the site-origin and contaminated samples.

**Figure 3:**
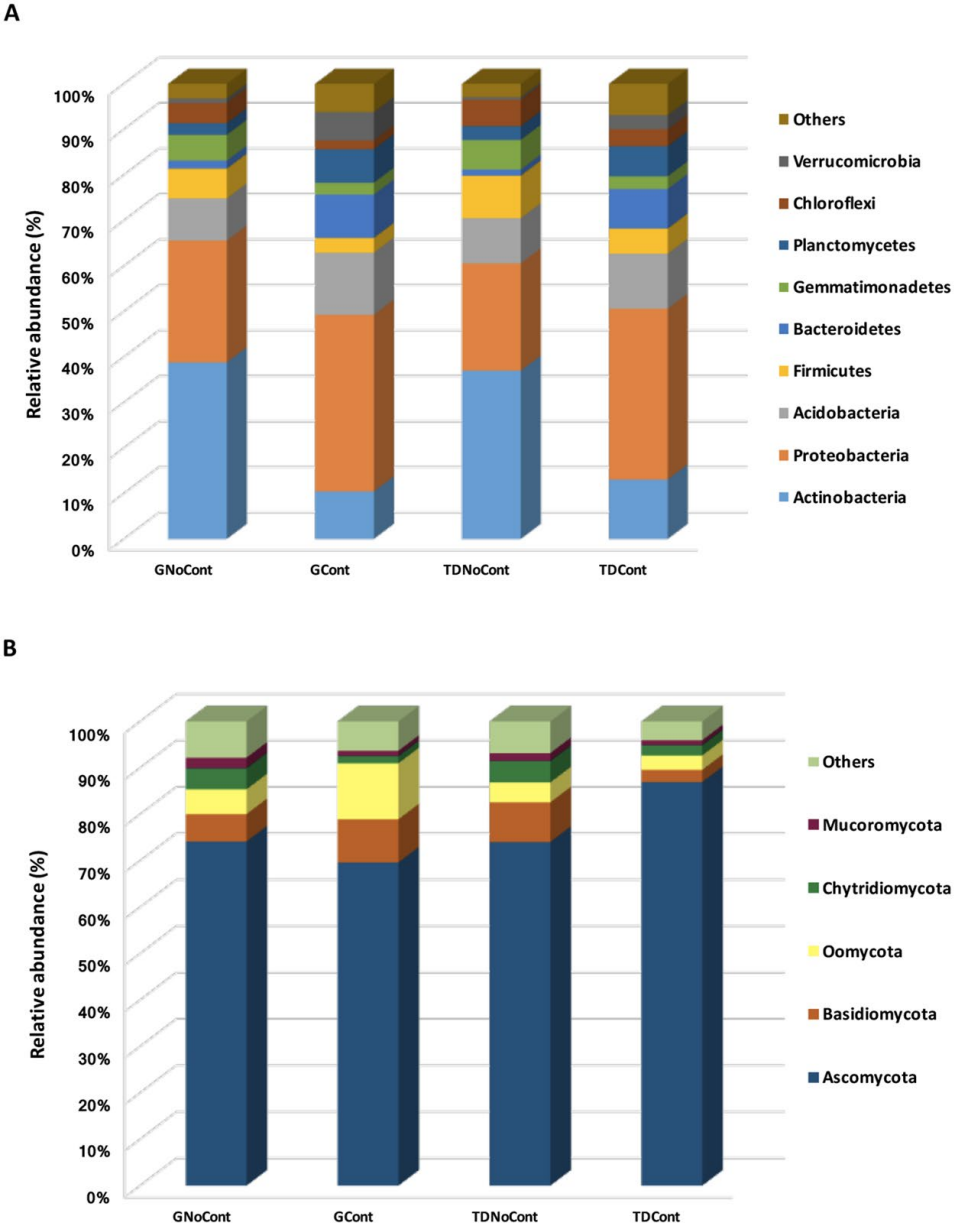
Abundance of prokaryotic (**A**) and eukaryotic (**B**) phyla in the soil samples at Giugliano and Trentola Ducenta site. Only OTUs with an incidence >1% in at least one sample are shown. GCont: contaminated soils at Giugliano site; GNoCont: noncontaminated soils at Giugliano site; TDCont: contaminated soils at Trentola Ducenta site; TDNoCont: noncontaminated soils at Trentola Ducenta site.

In total, forty-six different bacterial phyla were detected in the soil samples, but only Actinobacteria, Proteobacteria, Acidobacteria, Firmicutes, Bacteroidetes, Gemmatimonadetes, Planctomycetes, Chloroflexi and Verrucomicrobia were detected with an incidence >1% in at least one sample, accounting for approximately 93–97% of the total biodiversity in each sample (Fig. 3A). Although these taxa occurred in all samples, their abundance depended on the presence of pollutants, regardless of site origin. In particular, the relative abundance of Proteobacteria, Acidobacteria, Bacteroidetes and Verrucomicrobia was higher in contaminated soils (GCont and TDCont) than in noncontaminated soils (GNoCont and TDNoCont, Fig. 3A), highlighting their adaptation to this particular stress condition and a putative involvement in hypothetical organic xenobiotic compound degradation. Shahi *et al*.^25^ reported that these taxa were among the dominant phyla that significantly increased in petroleum hydrocarbon-contaminated soils and proved to be the most influential on the biodegradation of these pollutants. Moreover, consistent with previous studies, a shift to Proteobacteria dominance (from 26.7 to 38.8% and from 23.5 to 37.5% in G and TD, respectively) in hydrocarbon^26^ and heavy metal^27^ polluted soils was observed.

By contrast, the phyla Actinobacteria, Firmicutes, Gemmatimonadetes and Chloroflexi showed an opposite trend, strongly decreasing in contaminated soils (GCont and TDCont, Fig. 3A). The greatest reduction (approximately 3–4-fold) was observed for the Actinobacteria abundance (from 38.8% to 10.4% and from 37.0% to 13.0% in G and TD, respectively), while a decrease of approximately 2-fold was recorded for the relative abundance of Firmicutes, Gemmatimonadetes and Chloroflexi (Fig. 3A).

Although these taxa, especially Actinobacteria, are known to have specific hydrolytic enzymes for the decomposition of a wide variety of organic materials^28–30^ and are usually recovered in polluted environments^31^, their abundance may change significantly in association with contamination that could lead to shifts in pathways of fundamental biogeochemical processes^32^. Vaisvalavicius *et al*.^33^ noticed that a high contamination level reduced the counts and enzyme activity of some microbial groups (e.g., actinomycetes) with respect to uncontaminated soil, suggesting that they have low adaptability to contamination. Yin *et al*.^34^ reported that Actinobacteria were susceptible to heavy metals, and other studies showed that various bacterial populations, especially actinomycetes, were negatively correlated with metals, which drastically reduced their growth^35,36^.

As shown in Fig. 3B, the composition of eukaryotic populations in both sites was strongly dominated by Ascomycota (70–87%), the most abundant phylum among fungi recovered in aged polluted soils contaminated by both hydrocarbons (PAH) and heavy metals^7,37^ as well as vanadium^38^. Other eukaryotic phyla, such as Basidiomycota, Oomycota, Chytridiomycota and Mucoromycota, were found to a lesser extent. Their abundance varied as a function of site of origin (G or TD), and within each site, abundance was influenced by the presence of contaminants, except for Mucoromycota, which remained quite stable (approximately 1–2% in both sites, Fig. 2B). In particular, Basidiomycota ranged from 6% to 9% in GNoCont and GCont, respectively, while in TDNoCont and TDCont, its abundance was approximately 8.5% and 2.6%, respectively. Similarly, Oomycota abundance was approximately 5% and 12% in noncontaminated and soils in G, while its percentage was quite stable in TD soils (approximately 3–4%). These results confirmed that fungi were less affected by soil contamination than were bacterial populations^33,36^, as they have a wide variety of enzymes for degrading petroleum hydrocarbon pollutant^39^.

**Figure 4:**
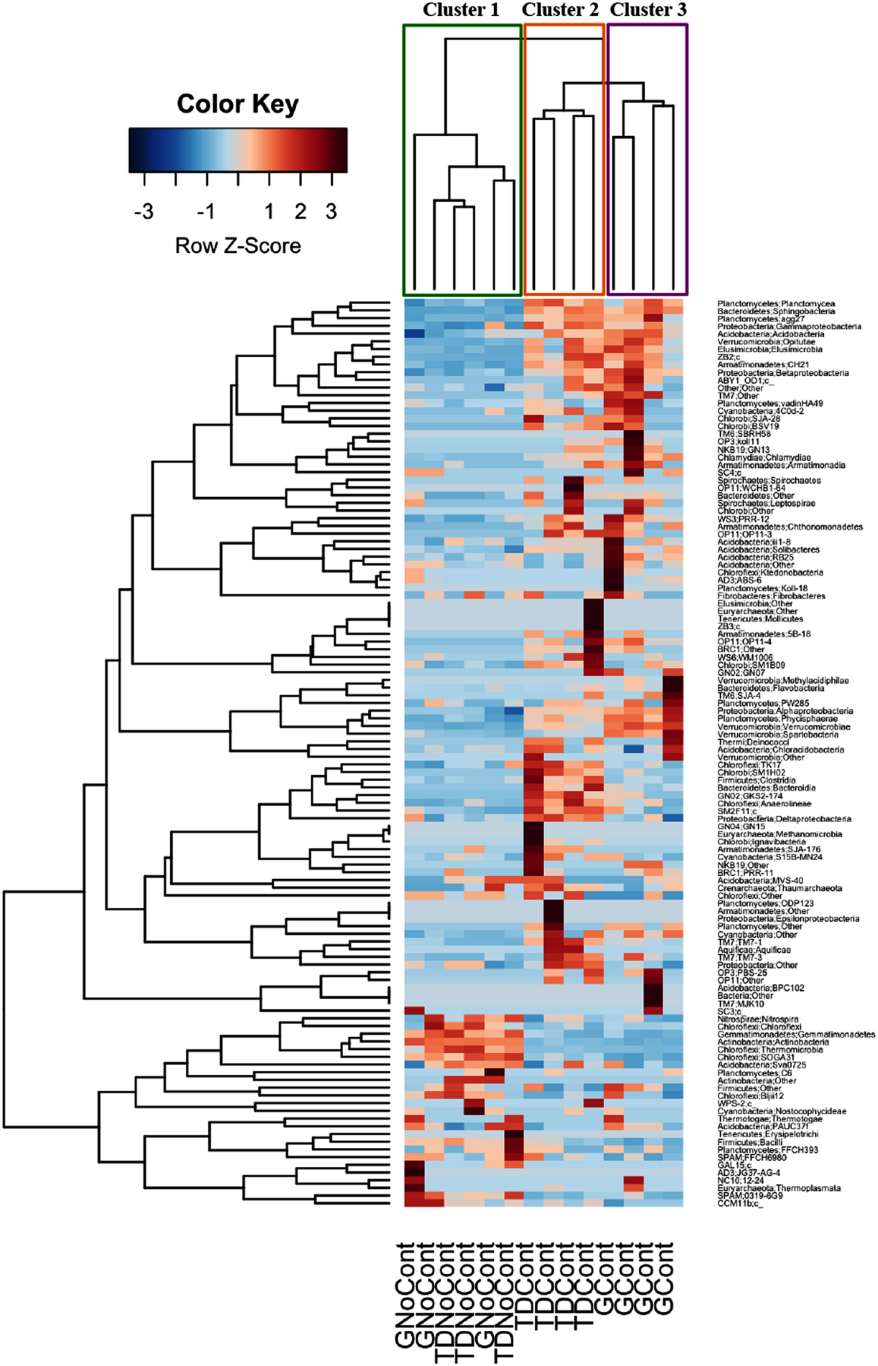
Heatmap representing prokaryotic taxa identified in Giugliano and Trentola Ducenta soil samples. Color scale indicates the relative abundance of each OTU within the samples. Dendrogram represents clustering patterns based on hierarchical clustering analysis on taxa and samples by Weighted Pair Group Method with Arithmetic Mean (WPGMA) method. GCont: contaminated soils at Giugliano site; GNoCont: noncontaminated soils at Giugliano site; TDCont: contaminated soils at Trentola Ducenta site; TDNoCont: noncontaminated soils at Trentola Ducenta site.

**Figure 5:**
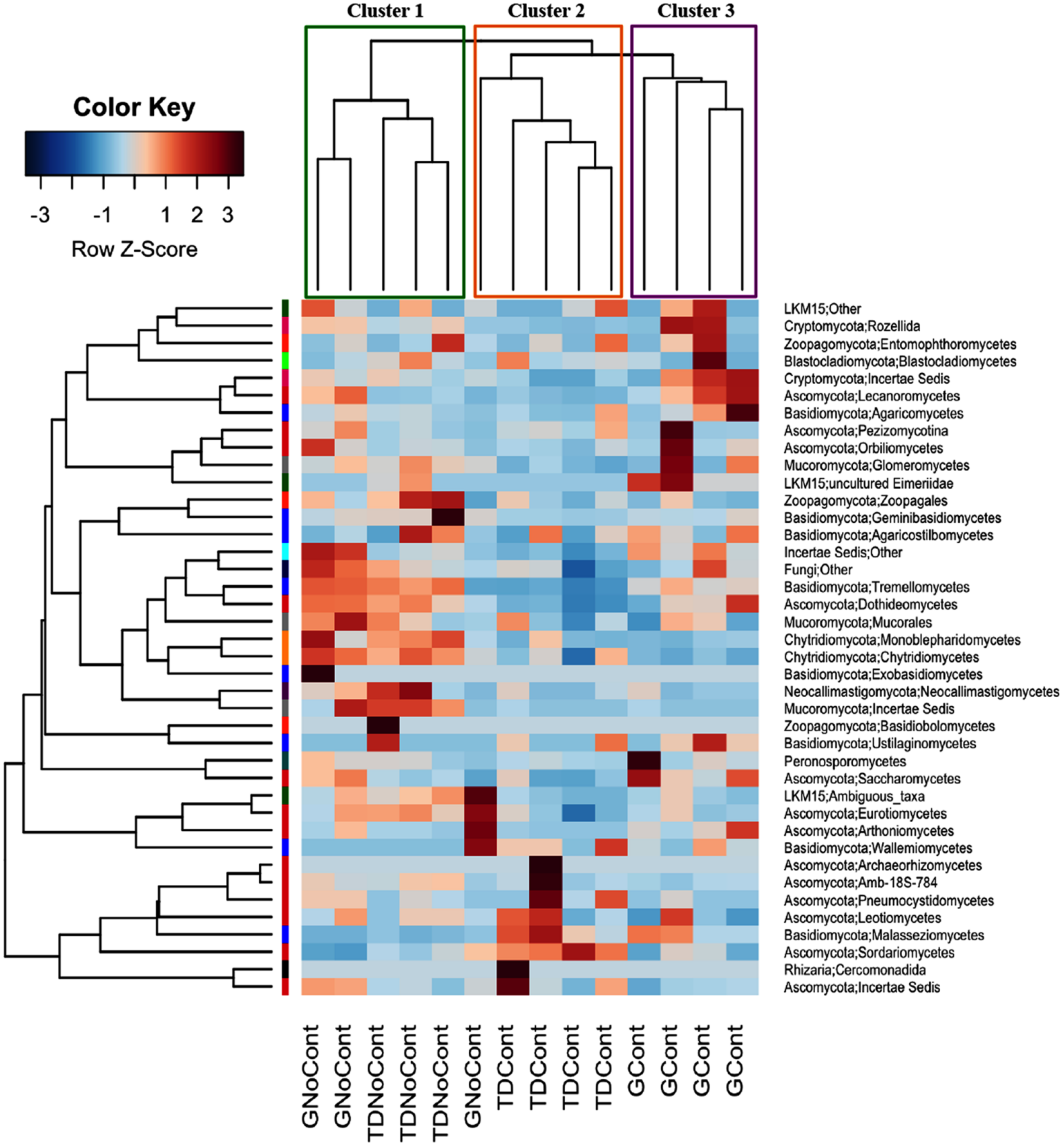
Heatmap representing eukaryotic taxa identified in Giugliano and Trentola Ducenta soil samples. Color scale indicates the relative abundance of each OTU within the samples. Dendrogram represents clustering patterns based on hierarchical clustering analysis on taxa and samples by Weighted Pair Group Method with Arithmetic Mean (WPGMA) method. GCont: contaminated soils at Giugliano site; GNoCont: noncontaminated soils at Giugliano site; TDCont: contaminated soils at Trentola Ducenta site; TDNoCont: noncontaminated soils at Trentola Ducenta site.

The microbial diversity was also analyzed at a deeper taxonomic level. The identification of OTUs at the class level is reported in the heatmap shown in Figs 4 and 5. As expected, the hierarchical clustering analysis based on taxa and samples grouped contaminated and noncontaminated soils. In detail, for bacterial communities, three major clusters were observed: noncontaminated GNoCont and TDNoCont samples (Cluster 1), TDCont samples (Cluster 2) and GCont samples (Cluster 3) (Fig. 4). Interestingly, Actinobacteria and Alphaproteobacteria exhibited the opposite trends in contaminated and noncontaminated soils. In particular, Actinobacteria was the dominant bacterial class in noncontaminated GNoCont and TDNoCont samples, accounting for approximately 39% and 37% of the total prokaryotic biodiversity, respectively, followed by Alphaproteobacteria (10–13%). By contrast, in contaminated GCont and TDCont soil samples, a significant increase was observed for Alphaproteobacteria, up to 21% and 18%, respectively, while Actinobacteria decreased to levels as low as 13–14% (Fig. 4). According to Kuppusamy *et al*.^40^. Alphaproteobacteria was the most abundant taxon in long-term contaminated soils compared to noncontaminated soil, suggesting that it plays an important role in the bionetwork function of these soils^27,41^.

In addition, Sphingobacteria abundance was markedly higher in contaminated samples (approximately 8%) than in noncontaminated soils (approximately 1%) (Fig. 4). Members of the class Sphingobacteria that have been recovered in polluted soil^41^ are reported to be involved in the degradation of aromatic and aliphatic hydrocarbons^42^.

Regarding eukaryotic populations, the hierarchical clustering analyses based on taxa and samples showed similar results, although the relative abundance of each prokaryotic class seemed to be specific to polluted environments (Fig. 5) in contrast to eukaryotic ones (Fig. 5). As shown in Fig. 5, Sordariomycetes was the most abundant class in all soil samples, accounting for approximately 50–70% of the total fungal biodiversity, confirming that this phylum was quite stable to environmental stress because it was dominant in both multicontaminated and noncontaminated ecosystems, as shown in previous studies^43,44^. Among the other eukaryotic classes recovered to a lesser extent, Eurotiomycetes and Chytridiomycetes decreased approximately 2-fold in contaminated soils, dropping to 6% and 4% and 1.5 and 2% in GCont and TDCont samples, respectively, compared to unpolluted soils (10% and 4% for Eurotiomycetes and Chytridiomycetes, respectively, in both sites). Dothideomycetes showed a similar trend, although a marked decrease was observed only in TDCont (2%) compared to TDNoCont (9%). Conversely, the abundance of Peronosporomycetes strongly increased in GCont (12%) compared to unpolluted GNoCont (5%), whereas its percentage remained quite stable in TD (3–4%, Fig. 5). The different responses to the contamination events of native bacterial and fungal populations, analyzed at the same taxonomic level, could be due to the lower sensitivity of fungi to any environmental changes because they generally show longer generation times than bacteria and therefore respond more slowly to soil perturbation^45^.

### Microbial biomarkers and core evaluation

**Figure 6:**
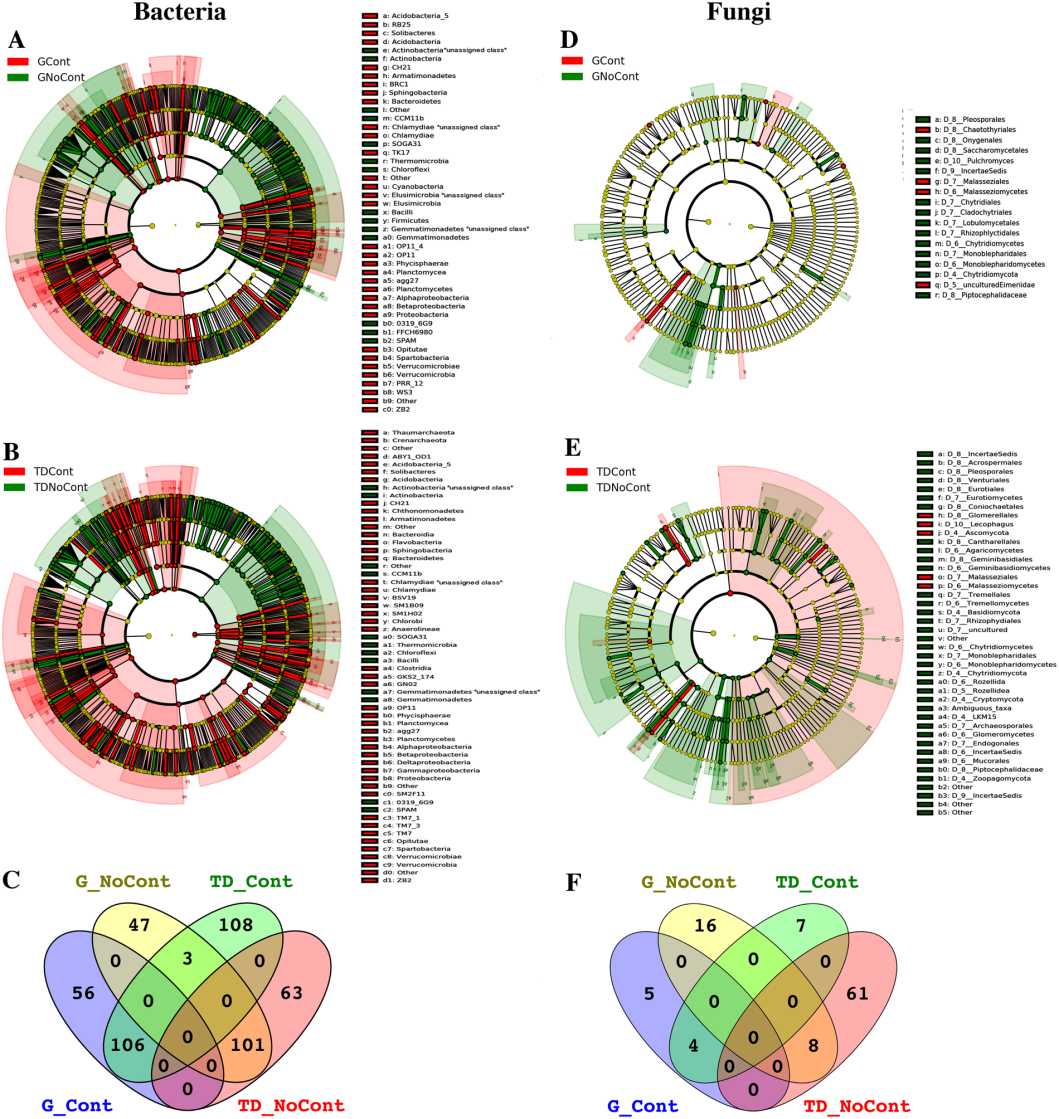
LEfSe cladograms showing taxa with different abundance values (LDA score >2; *p* < 0.05) in polluted (red) and unpolluted (green) soils of Giugliano and Trentota Ducenta sites. Central point represents root of the tree (Bacteria and Archaea in (**A**,**B**) plots; Fungi and Personosporomycetes in (**D**,**E**) plots), and each ring represents the next taxonomic level (phylum, class, family, genus and species). Red nodes represent taxa significantly overabundant in contaminated soils; green nodes represent taxa significantly overabundant in noncontaminated soils; nodes remaining yellow indicate taxa that were not significantly differentially represented (*p* > 0.05). (**C**,**F**) plots: Venn diagrams of shared prokaryotic (**C**) and eukaryotic (**F**) biomarkers among polluted and unpolluted soils in Giugliano and Trentola Ducenta sites. GCont: contaminated soils at Giugliano site; GNoCont: noncontaminated soils at Giugliano site; TDCont: contaminated soils at Trentola Ducenta site; TDNoCont: noncontaminated soils at Trentola Ducenta site.

Elucidating the responses of microbial communities to environmental stresses is fundamental to understanding the interactions between microorganisms and soil components and providing a possible approach for modeling pollution indicators. Biological factors, such as microorganisms, could indicate the environmental balance through biotic indexes derived from the observation of taxa. Therefore, in this study, LEfSe and Venn analyses were performed to identify specific bacterial or fungal populations as possible indicators of the health *status* of the soil. In particular, LEfSe analysis, through the detection of significant differences (LDA > 2; *P* < 0.05) in the abundance for different taxonomic rankings, allowed the identification of characteristic biomarkers of contaminated and noncontaminated soils at the sites TD and G. For the site G, the cladogram revealed 313 differential bacterial OTUs, of which 162 and 151 were detected in polluted and unpolluted soil, respectively (Fig. 6A; Supplementary Table S1), whereas only 33 differential fungal OTUs, 9 in contaminated and 24 in noncontaminated soils, were identified (Fig. 6D; Supplementary Table S2). As shown in Fig. 6B,E, a higher number of differential features was found in TD, represented by 381 bacterial (Supplementary Table S3) and 80 fungal (Supplementary Table S4) OTUs. Specifically, 217 bacterial and 11 fungal taxa were significantly overabundant when contamination was detected, while 164 bacterial and 69 fungal taxa were differentially abundant in unpolluted soil.

Since PCoA analysis on weighted UniFrac matrixes highlighted a strong phylogenetic diversity of the microbiota in contaminated soils influenced by the site-origin of samples, to identify potential bacterial and fungal biomarkers regardless of the site-origin of contaminated soil samples, Venn diagram analysis was carried out among biomarkers. As shown in Fig. 6, 106 bacterial taxa (Fig. 6C) belonging to 19 different classes (Acidobacteria_5, agg27, Alphaproteobacteria, Anaerolineae, Bacilli, Betaproteobacteria, CH21, Chlamydiae, Gammaproteobacteria, Opitutae, Phycisphaerae, Planctomycea, PRR_12, Solibacteres, Spartobacteria, Sphingobacteria, TM7_3, Verrucomicrobiae, and ZB2; Supplementary Table S5) and 4 fungal taxa (Fig. 6F) belonging to only one order (Malasseziales; Supplementary Table S6) were shared between GCont and TDCont soils. Among these taxa, the most significant bacterial biomarkers (LDA > 4; *P* < 0.05) were represented by Alphaproteobacteria, Betaproteobacteria, Planctomycea and Sphingobacteria. Proteobacterial communities, mainly composed of Alpha-, Beta- and Gammaproteobacteria, might be involved in the biodegradation or biotransformation of numerous organic compounds, although their proportions varied among different polluted environments^7,46^. An interesting finding was that all OTUs classified as Betaproteobacteria in polluted soils were identified as methylotrophic bacteria, which are known to have great potential in the bioremediation of environmental pollutants such as chlorinated solvents and methyl tert-butyl ether (MTBE)^47^ as well as PAHs^6,48^.

Members of the phylum Planctomycetes were reported to be highly correlated with high copper and lead concentrations in contaminated soils^7^ and to rank among the top taxa in oil-contaminated soil^49^. In addition, OTUs identified as Sphingobacteria and Acidobacteria were other bacterial biomarkers recovered in polluted soils. These taxa were previously recovered from plant biomass-degrading microorganisms ecologically related to the soil ecosystem^10,50–52^, highlighting their ability to synthetize a wide number of enzymes for the depolymerization of recalcitrant organic matter. Moreover, unculturable bacteria, such as candidate division agg27, CH21, TM7_3 and ZB2, emerged as new biomarkers of polluted soils. In fact, among taxa that drive the biodegradation of hydrocarbons in natural soils, uncultured bacteria could play a key role^8^. The other bacterial biomarkers of polluted soils were identified as Anaerolineae, Bacilli, Chlamydiae, Opitutae, Phycisphaerae, Spartobacteria and Verrucomicrobiae. Sutton *et al*.^5^ reported that Anaerolineae was associated with anaerobic degradation of oil-related compounds and that its presence in soils could be related to the natural attenuation under anoxic conditions. Verrucomicrobia was recognized as the most abundant phylum in an oil-contaminated soil sampled from major oilfields in Northern China^49^, and it was ubiquitous in multiple petroleum-contaminated soils^25,53^. Finally, among eukaryotes, Basidiomycota, particularly Malasseziales, could be considered a fungal biomarker in contaminated soils. Although species belonging to this taxon remain uncultured, they were previously recovered at the later stages of the composting process of recalcitrant materials^54^ as well as from deep-sea extreme environments^55–57^.

In unpolluted soils, 101 bacterial taxa belonging to 9 classes (Fig. 6C; Supplementary Table S5) and 8 fungal taxa belonging to 4 orders (Fig. 6D; Supplementary Table S6) were shared between the two sites. Interestingly, among these taxa, Actinobacteria was the most abundant, accounting for 58% of OTUs identified as biomarkers when no contamination was detected. Although several works reported that members belonging to the Actinobacteria class can synthesize enzymes able to degrade a high variety of xenobiotic organic compounds^58–61^, the results obtained in this work showed that they are sensitive to stress conditions due to the presence of high multicontamination in a complex natural environment, such as soil. Dias *et al*.^62^ reported that a large amount of fresh diesel in a contaminated soil led to unfavorable environmental conditions for the growth of Actinobacteria, which increased only at the final stage of the bioremediation process, when the levels of available hydrocarbons were low. In addition, a wide variety of species found within Actinobacteria highlighted that under unstressed conditions, no selection pressure occurred at either site.

In conclusion, this study has important implications for the microbial ecology of soil belonging to the Regional and National Interest Priority Sites. Even if the response of the microbial communities to environmental stresses is a critical issue in soil ecology, our results showed that microbial population composition shifted significantly in contaminated environments. The microbial populations were sensitive to pollution, since the relative abundance of different taxa strongly changed, establishing a new prokaryotic and eukaryotic community in the contaminated soils. In addition, potential biomarkers that could be used as indicators for the ecological *status* and health of agricultural soils as well as for possible biotechnological applications were identified. In particular, Actinobacteria represents a sensitive biomarker for assessing soil pollution and therefore could provide general information on the health *status* of the environment. This approach could also be useful for bioremediation purposes to identify autochthonous populations for isolating microbial degraders from contaminated soils with specific xenobiotic toxic compounds. Therefore, this work improves the knowledge about the responses of indigenous microbial populations to anthropogenic activities, particularly related to the potential ecological risks of contamination in arable lands and the capacity of natural ecosystems to develop a microbiota adapted to polluted soil. Such information cannot be ignored in the evaluation of the adverse effects of potentially toxic metal distribution and accumulation trends in agricultural soil. Ecological risks, along with the more commonly assessed human health risks, could aid in strategic planning and management aimed at reducing soil contamination.

Investigating the microbiota of multicontaminated agricultural soils could represent a good opportunity to clarify microbial adaptation with important applications in the field of bioremediation and/or biostimulation^11^ and to understand the capacity of microbial populations to colonize the soil ecosystem to recover natural biofertility. In fact, evolutionary studies would not only improve the knowledge of the microbial community ecology in contaminated environments but also allow the implementation of potential bioremediation strategies to address the increasing impacts of xenobiotics in the ecosystems^63^. However, further studies are needed to determine the resistance/tolerance and molecular mechanisms involved in the adaptation of spontaneous microbial biodegraders to contaminated and noncontaminated agricultural soils.

## Methods

### Study sites and soil sampling

The study sites were two fallow rural fields that were used in the past for illegal waste disposal and dumping: Giugliano-NA and Trentola Ducenta-CE. They were used as pilot fields in the LIFE-Ecoremed project aimed to validate eco-compatible techniques for soil remediation^64^. The two study sites (Giugliano, G: 40.960499 N, 14.118677 E and Trentola Ducenta, TD: 40.966496 N, 14.147811 E) were located 3 km apart in a potentially contaminated plain of Campania region^19^ and therefore characterized by Mediterranean climatic conditions (rainy/temperate autumn-winter and arid/ warm spring-summer).

After waste removal, soils were sampled on December 2013 under the following environmental conditions (monthly values): 23, 10and 16 °C for maximum, minimum and mean air temperature, 80% for mean air relative humidity and a cumulative rainfall of 71 mm^65^. The soil samples were analyzed for heavy hydrocarbons (C > 12), several PAHs and PTEs. Details of the soil sampling scheme based on a two-level grid resolution to assess pollutant spatial distribution are described by Monaco *et al*.^66^ and Rocco *et al*.^67^. The Giugliano site was mainly polluted by heavy hydrocarbons (71% of the area), Cu (22% of the total area) and Zn (12% of the area); the Trentola-Ducenta site was polluted by heavy hydrocarbons (78% of the total area) and Cu (7% of the area)^64,66–68^. Based on these results and taking into account the spatial distribution of pollution, 14 plots (7 in Giugliano and 7 in Trentola-Ducenta) with different pollution levels were selected for microbial analysis. Form these plots, 1 kg soil samples were collected, homogenized and sent to the microbiological laboratory.

### Chemical analysis

Soil samples were analyzed for main chemical properties (pH, organic carbon content, cation exchange capacity, carbonate content) and particle size analysis. The pseudodototal content of 13 PTEs was measured using microwave-assisted acid digestion in *aqua regia* followed by inductively coupled plasma-atomic emission spectrometry (EPA 3051 A and EPA 6020 A). Heavy hydrocarbons (C > 12) were determined according to UNI EN ISO 16703. For PAH determination, the US-EPA method 8270D was applied with a gas chromatograph coupled to a quadrupole mass spectrometer (GC/MS). In Italy, soil quality standards for agricultural areas have not yet been established. Therefore, all data were referenced against the threshold limits imposed by the Italian Action Levels for Residential land use (IALR) established under Italian environmental law (D.Lgs 152/2006) and the local Soil Baseline Reference values^69,70^.

More details on the soil analysis and pollutant extraction methods are reported by Monaco *et al*.^66^ and Rocco *et al*.^67^.

### DNA extraction and high-throughput sequencing

Total genomic DNA was extracted using a FastDNA SPIN Kit for Soil (MP Biomedicals, Illkirch Cedex, France) according to the manufacturer’s instructions.

The microbial diversity was evaluated by amplicon-based metagenomic sequencing using the primers S-D-Bact-0341F50 (5′-CCTACGGGNGGCWGCAG-3′) and S-D-Bact-0785R50 (5′-GACTACHVGGGTATCTAATCC-3′)^71^ for the bacterial V3-V4 region of the 16S rRNA gene and the primers NS1 (5′-GTAGTCATATGCTTGTCTC-3′) and NS2 (5′-GGCTGCTGGCACCAGACTTGC-3′)^72^ for the fungal 5′-end of the 18S rRNA gene. Amplicon purification, multiplexing and sequencing were carried out by Genomix4 Life s.r.l. (Salerno, Italy) as reported in the Illumina Metagenomic Sequencing Library Preparation manuals. Sequencing was carried out on a MiSeq platform (Illumina Italy s.r.l., Milan, Italy), leading to 250 bp or 300 bp paired-end reads for bacteria and fungi, respectively.

### Bioinformatics and data analysis

Row reads were quality analyzed and filtered using PRINSEQ.^73^. Low-quality reads (Phred score <20) were trimmed, and reads shorter than 60 bp were discarded in end-to-end, sensitive mode.

Paired-end reads were merged using FLASH^74^, and sequences were then analyzed using QIIME 1.9.1 software^75^. Operational taxonomic units (OTUs) at 97% sequence identity were picked through a *de novo* approach, and the uclust method and taxonomic assignment were obtained using the RDP classifier and the Greengenes^76^ or SILVA SSU/LSU^77^ database for 16S and 18S rRNA sequences, respectively. Chloroplast contamination was removed from 16S OTU tables, while only Fungi and Peronosporomycetes sequences were retained in 18S OTU tables. Singletons were also discarded from filtered OTU tables, and the relative abundance of other taxa was recalculated. To avoid biases due to different sequencing depths, OTU tables were rarefied at the lowest number of sequences per sample.

Statistical analyses and plotting were carried out in R (http://www.r-project.org). A hierarchical clustering heatmap was obtained through the made4 package using the weighted pair group method with arithmetic mean (WPGMA) method. Alpha (Chao1 richness and Shannon diversity indexes) diversity analysis was computed by QIIME on rarefied OTU tables, and the pairwise Wilcoxon test was used in R to test for significant differences. Beta diversity was calculated and plotted using the phyloseq R package^78^ for the Weighted UniFrac, and ANOSIM statistical analysis was then carried out with the Vegan R package^79^ on the same distance matrix.

Linear discriminant analysis (LDA) effect size (LEfSe)^80^ was used to identify differentially abundant OTUs for a *P* < 0.05 after Kruskal-Wallis test between groups. The results are ranked by the effect size analysis and represent discriminative features with an LDA score >2.0.

### Accession codes

The raw Illumina sequencing data are available in the Sequence Read Archive database of the National Center of Biotechnology Information (SRP129887).

## Electronic supplementary material

Dataset 1
